# Non-intubated tracheal resection and reconstruction for a tracheal tumor in an 8-year-old child

**DOI:** 10.1186/s13019-024-02949-8

**Published:** 2024-07-26

**Authors:** Yaoliang Zhang, Zhongqiao Mo, Chao Yang, Jianxing He, Shuben Li, Lan Lan

**Affiliations:** 1 Department of Anesthesiology, the First Affiliated Hospital of Guangzhou Medical University, No. 151, Yanjiang Xi Road, Guangzhou, Guangdong People’s Republic of China; 2 Department of Thoracic Surgery, The First Affiliated Hospital of Guangzhou Medical University, No. 151, Yanjiang Xi Road, Guangzhou, Guangdong People’s Republic of China; 3 National Clinical Research Center for Respiratory Disease, State Key Laboratory of Respiratory Disease, Guangzhou Institute of Respiratory Health, the First Affiliated Hospital of Guangzhou Medical University, Guangzhou, China

**Keywords:** Non-intubated, Trachea resection, Tracheal tumors, Children

## Abstract

**Introduction:**

It has been reported that non-intubated anesthesia can be used successfully in adult trachea reconstruction. Herein, our center reported a case of a child undergoing non-intubated trachea reconstruction for benign tracheal tumors.

**Case description:**

In January 2023, it was decided to attempt tracheal resection and reconstruction (TRR) in an 8-year-old child with an inflammatory myofibroblastic tumor under non-intubated spontaneous breathing. After anesthesia induction, the laryngeal mask airway (LMA) was inserted. Thereafter, a bilateral superficial cervical plexus block was performed with 15 mL of 0.25% ropivacaine injected into each side. The patient was induced to resume spontaneous breathing by artificially assisted ventilation with an oxygen flow of 2 to 5 L/min and FiO_2_=1. After tracheotomy, the oxygen flow was increased to 15 L/min to improve the local oxygen flow to maintain the pulse oxygen saturation (SpO_2_) above 90% under spontaneous breathing. The patient had stable spontaneous breathing after tracheal anastomosis. The anastomosis was perfect without leakage. The LMA was removed and oxygen was given by the nasal catheter under light sedation at post anesthesia care unit (PACU).

**Conclusion:**

Tracheal reconstruction under spontaneous breathing may be an alternative anesthesia method for upper tracheal surgery in children.

**Supplementary Information:**

The online version contains supplementary material available at 10.1186/s13019-024-02949-8.

## Introduction

Tracheal resection and reconstruction (TRR) is an effective treatment for tracheal tumors [1], especially for pediatric tracheobronchial inflammatory myofibroblastic tumor [2]. However, it poses a huge challenge for anesthesiologists to share the airway with the surgeon during the tracheal reconstruction. Traditional TRR applies endotracheal intubation and surgical cross-field intubation under general anesthesia [3]. In recent years, non-intubated spontaneous breathing has been widely used in video-assisted thoracoscopic surgery (VATS) [4–6], and has also been successfully applied in TRR [7–10]. Until now, the application of non-intubated in pediatric TRR is seldom reported. Herein, it was reported a case of the world’s youngest child undergoes non-intubated TRR for benign tracheal tumors.

## Case description

An 8-year-old boy (134 cm in height and 45 kg in weight) came to our hospital with the diagnosis of an inflammatory myofibroblastic tumor. Two months ago, the child developed a cough, wheezing, and polypnea, which worsened after activity. A mass was found in the upper trachea with a computerized tomography (CT) scan, which blocked the main airway. Bronchoscopy, tracheobronchial tumor ligation, and excision were performed to relieve the symptoms and clarify the tumor pathology. However, the electronic bronchoscope reexamination showed that local tumor tissues still lay in the endotracheal and invaded the whole layer of the tracheal wall. Therefore, the patient and his family decided to accept TRR.

Non-intubated TRR was decided to apply after a multidisciplinary discussion. The anesthesia induction was propofol (0.5 mg/kg), atropine (0.01 mg/kg), midazolam (0.05 mg/kg), sufentanil (0.1 µg/kg), and esketamine (0.5 mg/kg). When bispectral index (BIS) < 60, the supreme laryngeal mask airway (LMA) was inserted and connected to the ventilator with synchronous intermittent instruction ventilation (SIMV). The gastric tube is inserted through the LMA. A bilateral superficial cervical plexus block was performed under ultrasound guidance with 15 mL 0.25% ropivacaine injected into each side. The anesthesia was maintained with dexmedetomidine 0.5 ∼ 1.0 µg/kg/h, remifentanil 0.03 ∼ 0.05 µg/kg/min, and propofol 3 ∼ 5 mg/kg/h. The depth of anesthesia maintained BIS value between 40 and 60. The glottis and tracheal were blocked with 2% lidocaine spraying through fiberoptic bronchoscope and repeated every 60 min.

**Fig. 1:**
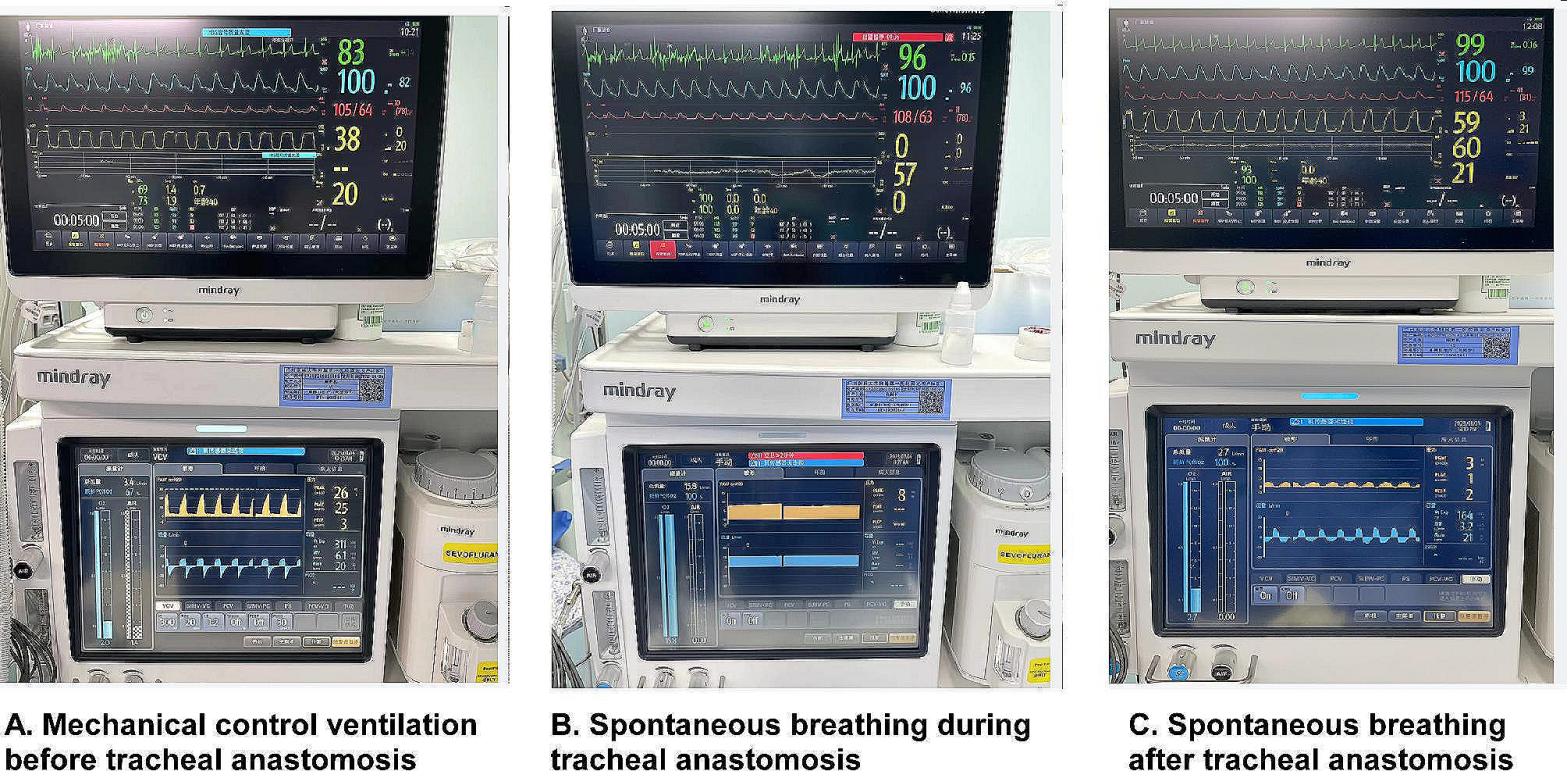
Vital signs and respiratory parameters

**Fig. 2:**
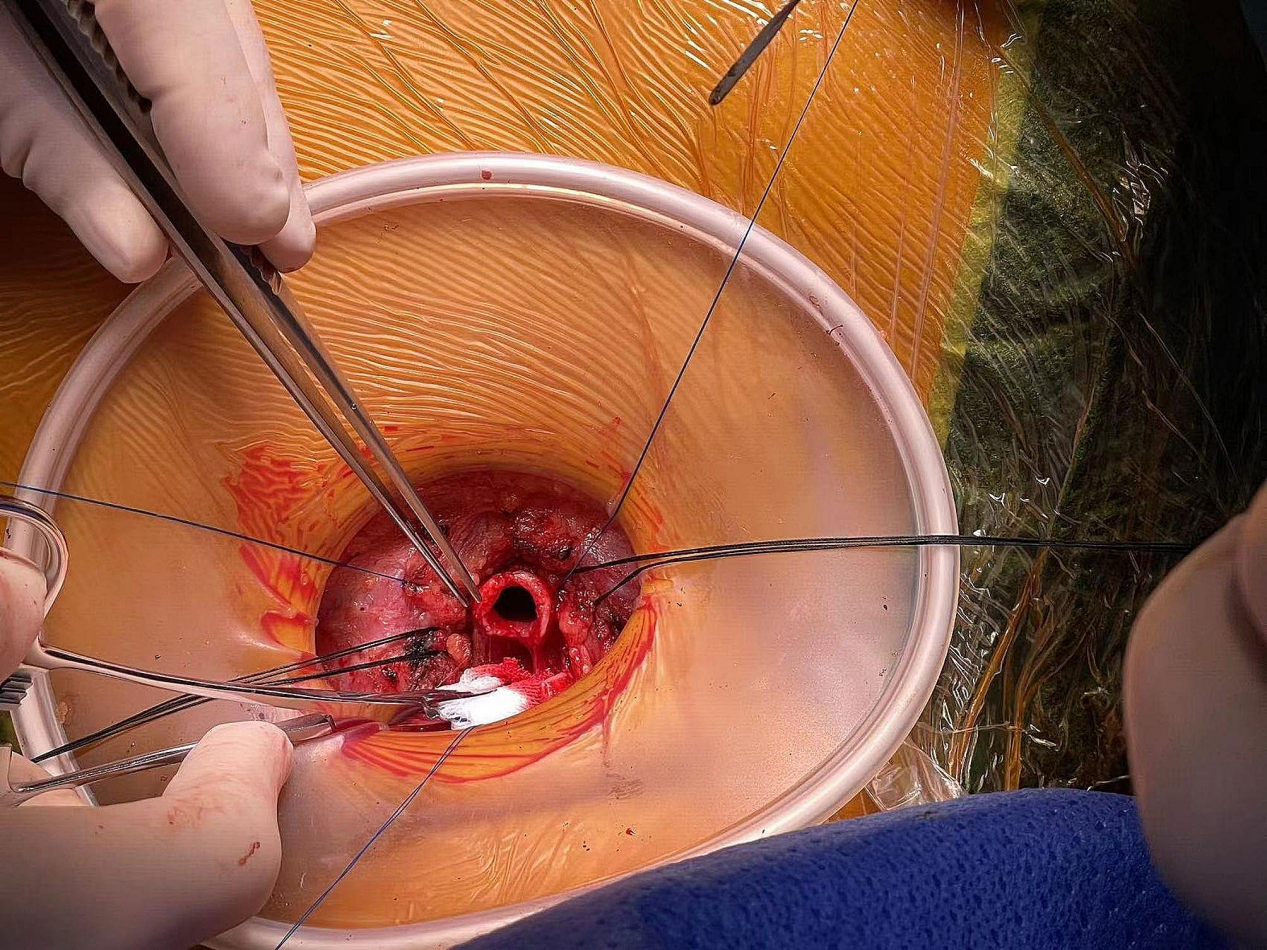
Tracheal transaction

**Fig. 3:**
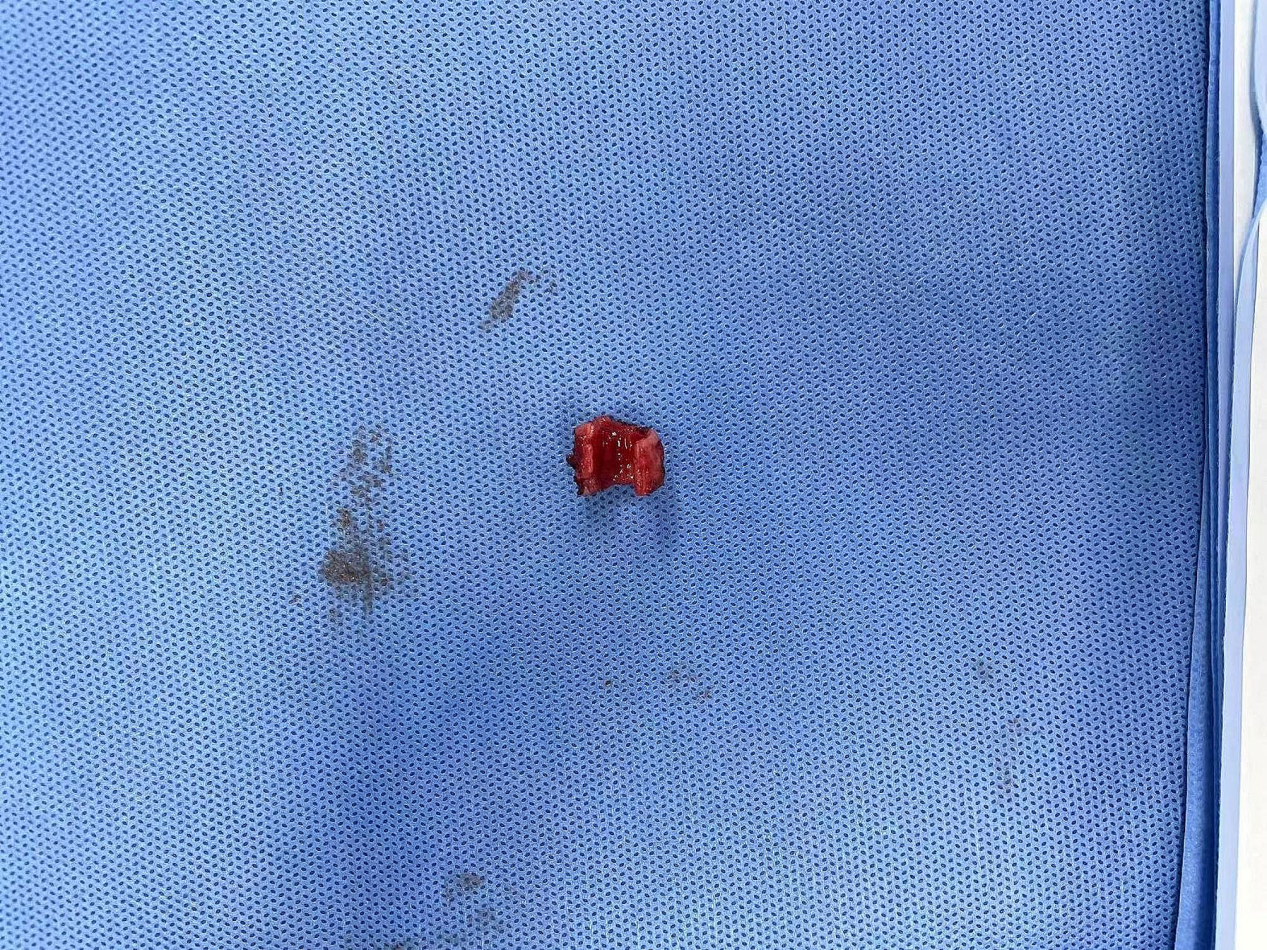
The excised part of the trachea

The preoperative electronic bronchoscopy showed that the tumor was located in the cervical trachea with a length of about 1.2 cm, 2.5 cm under the vocal cord, and 4 cm above the carina. It was made a 5 cm long low-necked incision and dissected the trachea layer by layer. The patient was induced to resume spontaneous breathing by changing the ventilation mode from SIMV to artificially assisted ventilation with an oxygen flow of 2 to 5 L/min and FiO_2_=1. After tracheotomy, the oxygen flow was increased to 15 L/min to improve the local oxygen flow to maintain the SpO_2_ above 90% under spontaneous breathing during anastomosis (Fig. 1, video 1 and video 2). The margin was 0.5 cm away from the tumor, and the trachea lesion was removed about 2 cm, and then an end-to-end anastomosis of the trachea was performed (Figs. 2 and 3).

The child had stable spontaneous breathing and vital signs during tracheal anastomosis and was sent to the post anesthesia care unit (PACU) immediately after the operation (Table 1). The total surgical time was 255 min, and the duration of end-to-end anastomosis was 30 min. The LMA was removed and oxygen was given through the nasal catheter under light sedation. Finally, the patient was discharged from the hospital in postoperative 9 days.

**Table 1:** Intraoperative vital signs and blood gas analysis

Varialbes	HR (b/time)	MAP (mmHg)	pH	paCO_2_ (mmHg)	paO_2_ (mmHg)	SaO_2_ (%)
Before resection of tracheal mass	88	80	7.313	50.9	409	100
During tracheal anastomosis	92	80	7.115	71.7	367	100
After tracheal anastomosis	89	81	7.352	36.5	94.6	97

## Discussion

TRR is a difficult technique that requires close cooperation and coordination between surgeons and anesthesiologists. When the airway is open, a smaller sterile tracheal tube is inserted into the distal airway and connected to an external ventilator to acquire respiratory support. The obvious limitations of the traditional method are first that the surgical cross-field intubation interferes with surgical procedures and makes end-to-end anastomosis difficult. Secondly, muscle relaxation may aggravate the airway obstruction, and even complete airway occlusion, requiring emergency rescue [11]. Finally, repeated intubation will cause tumor tissue breaking off and seeding in the trachea. Non-intubated TRR can remove the tumor lesion and reconstruct the trachea efficiently due to no tracheal tube [9, 12]. In addition, nerve integrity is easy to be tested under spontaneous breathing.

Non-intubated TRR is performed under the assistance of bilateral superficial cervical plexus block or epidural anesthesia combined with intravenous anesthesia, and LMA is inserted to ensure the patency of the upper airway [13]. The patient resumed spontaneously breathing before tracheotomy. After that, a 10 F hollow hard catheter was inserted into the distal trachea under spontaneous breathing with an external oxygen flow rate of 5–10 L/min, to increase the inhalation oxygen flow and maintain SpO_2_ > 90%. If necessary, a 10 F hollow catheter was connected to the high-frequency jet ventilation (driving pressure = 0.5-1.0 bar, FiO_2_ = 1.0, respiratory rate = 60 times/min, I: E ratio 1:1). If SpO_2_ did not improve significantly, the remedy measures of distal tracheal intubation were performed. In a retrospective case series at our center, Zhou et al. reported [7] that a total of 51 adult patients have successfully performed tracheal reconstruction under satisfactory spontaneous breathing. This experience also contributes to the implementation of spontaneous breathing in pediatric TRR.

Due to the small trachea and small inner diameter of the tracheal tube, good ventilation cannot be performed in TRR under traditional methods, resulting in high airway pressure, carbon dioxide accumulation, postoperative mechanical ventilation-related complications, etc. Extracorporeal circulation is sometimes required during the operation. Poor compliance in children and discomfort caused by postoperative tracheal intubation result in children having to maintaining sedation and analgesia for a longer time, which delays rehabilitation. Non-intubated VATS has been reported in children with fast full awakening, less time in PACU, and a lower incidence of emergency psychosis [14]. Median time to oral feeding, activity, and pain intensity were also significantly reduced. Non-intubation is a viable strategy for tracheal surgery in highly selected patients. Jiang et al. reports [15] that the duration of the operation and the trachea anastomosis, and the stay at the postoperative hospital are significantly shorter in the non-intubation group.

Based on the experience of non-intubated adults TRR [7, 9, 15, 16], it was decided to perform a non-intubated method for this child. Preoperative enhanced CT and 3D airway reconstruction helped to assess the airway and tracheal involvement of the tumor. Preoperative bronchoscopy was helpful for accurately locating the lesion, which was essential for planning surgery and anesthesia. The LMA under intravenous anesthesia combined with a bilateral superficial cervical plexus block was used. The patient resumed spontaneous breathing approximately 15 min before tracheotomy. After tracheotomy, a 10 F hollow hard catheter was no longer used to deliver oxygen to the distal trachea, but the oxygen flow rate was adjusted to 15 L/min, the oxygen concentration was 100%, the APL valve was closed, and oxygen was continuously supplied to the tracheal section through LMA to improve the local oxygen concentration, which was different from the scheme of non-intubated adult TRR [7, 9]. During the operation, it was observed that the SpO_2_ remained above 96%. This not only maintained a good oxygen supply but also made the operating field completely free of tracheal tube interference, significantly improving the speed and quality of end-to-end anastomosis. Of course, remedy resuscitations had been prepared once the SpO_2_ could not be maintained. Firstly, the high-flow nasal ventilator was ready, which can provide a maximum oxygen flow of 80 L/min in the case of weak breathing, to deliver more oxygen into the trachea section and the alveoli for gas exchange and maintenance of oxygenation [17, 18]. In addition, high-frequency jet ventilation is also an ideal choice. Secondly, emergency endotracheal intubation across the operative field can be carried out when SpO_2_ decreases further. Finally, extracorporeal membrane oxygenation is the last straw when all methods to improve oxygenation fail to work. Intraoperative vital signs and arterial blood gas (ABG) were listed in Table 1, and ABG was performed every 20 min after tracheostomy.

Postoperative care is also important. The patient had to be fixed in the position of bowing his head and bending his neck. It was concerned that the patient would experience violent agitation while awake, leading to anastomotic tear, since poor compliance in younger children, combined with uncomfortable immobilization and the LMA irritation. Therefore, it was decided to continue intravenous infusion with dexmedetomidine (0.5 µg/kg/h) and remifentanil (0.03 µg/kg/min) during resuscitation, maintaining a state of mild sedation and analgesia. Finally, the child recovered calmly and cooperatively, without crying or agitation, and had stable spontaneous breathing. The LMA was removed and oxygen was administered through a nasal cannula. The visual analog scale score was 3, and the Ramsay score was 2. He was safely returned to the ward accompanied by his parents.

## Conclusion

Tracheal reconstruction under spontaneous breathing may be also an alternative anesthesia method for upper tracheal surgery in children, which is feasibility and effective. It still requires multicenter to participate and include a large number of samples to determine the benefits of non-intubated TRR for children.

### Electronic supplementary material

Below is the link to the electronic supplementary material.

Supplementary Material 1

Supplementary Material 2

Supplementary Material 3

## Data Availability

No datasets were generated or analysed during the current study.
